# Altered Metabolomic Profile in Patients with Peripheral Artery Disease

**DOI:** 10.3390/jcm8091463

**Published:** 2019-09-14

**Authors:** Ahmed Ismaeel, Marco E. Franco, Ramon Lavado, Evlampia Papoutsi, George P. Casale, Matthew Fuglestad, Constance J. Mietus, Gleb R. Haynatzki, Robert S. Smith, William T. Bohannon, Ian Sawicki, Iraklis I. Pipinos, Panagiotis Koutakis

**Affiliations:** 1 Department of Nutrition, Food and Exercise Sciences, Florida State University, Tallahassee, FL 32306, USA; 2 Department of Environmental Science, Baylor University, Waco, TX 76798, USA; 3 Department of Surgery, University of Nebraska at Medical Center, Omaha, NE 68198, USA; 4 Department of Biostatistics, University of Nebraska Medical Center, Omaha, NE 68198, USA; 5 Department of Surgery, Baylor Scott and White Hospital, Temple, TX 76508, USA

**Keywords:** peripheral artery disease (PAD), metabolomics, claudication, critical limb ischemia (CLI)

## Abstract

Peripheral artery disease (PAD) is a common atherosclerotic disease characterized by narrowed or blocked arteries in the lower extremities. Circulating serum biomarkers can provide significant insight regarding the disease progression. Here, we explore the metabolomics signatures associated with different stages of PAD and investigate potential mechanisms of the disease. We compared the serum metabolites of a cohort of 26 PAD patients presenting with claudication and 26 PAD patients presenting with critical limb ischemia (CLI) to those of 26 non-PAD controls. A difference between the metabolite profiles of PAD patients from non-PAD controls was observed for several amino acids, acylcarnitines, ceramides, and cholesteryl esters. Furthermore, our data demonstrate that patients with CLI possess an altered metabolomic signature different from that of both claudicants and non-PAD controls. These findings provide new insight into the pathophysiology of PAD and may help develop future diagnostic procedures and therapies for PAD patients.

## 1. Introduction

Peripheral artery disease (PAD) is an atherosclerotic condition of the arteries supplying the lower extremities. PAD affects over 200 million people around the world, with an estimated prevalence of more than 20% for individuals over 80 years old [1,2]. The most common manifestation of symptomatic PAD is intermittent claudication (IC), a painful discomfort in the leg muscles during walking that produces gait dysfunction and severe functional limitation. A small subset of PAD patients (approximately 1.3%) present with critical limb ischemia (CLI), the more severe form of PAD, manifested by ischemic rest pain and tissue loss/gangrene [3,4]. Work from several groups, including our own, has demonstrated significant degenerative changes in all the tissues of the chronically ischemic legs of patients with PAD, including skin, muscles, nerves, and subcutaneous tissues [5,6]. These changes have been best studied in the skeletal muscle of the affected limbs [7,8,9] and demonstrate an acquired myopathy with significant metabolic components. The biochemical characteristics of this myopathy include mitochondrial dysfunction, accumulation of metabolic intermediates, increased oxidative damage, and cytokine upregulation [1,5,6,10,11,12,13,14]. These metabolic myopathic changes are present in the legs of both IC and CLI patients, with the myopathy of CLI being more severe than that of IC [15]. Beyond this ischemic myopathy, PAD is also directly associated with conditions like dyslipidemia, obesity or cachexia, diabetes, and insulin resistance, all of which involve dysregulation of metabolism and energy homeostasis [16,17].

Metabolomics, the study of small-molecule metabolites in biological systems [18,19], has been increasingly applied to cardiovascular disease, leading to recent discoveries in disease-specific biomarkers and their mechanistic implications [20]. Metabolomics can detect, quantify, and identify a number of intermediate compounds and end products of cellular metabolism in body fluids, tissues, and cells, thus providing a molecular phenotype that directly reflects biochemical activity [21,22]. This strategy can therefore be useful in identifying the signature profiles of patients at different stages of a disease, and has the potential of improving our understanding of the pathogenesis, diagnosis, risk-stratification, monitoring of disease progression, personalization of treatment [23], and monitoring of the response to different therapies [24].

The clinical application of metabolomics in the study of PAD has been thus far explored with two studies evaluating near-term mortality and arterial stiffness in PAD patients. The first study showed that the ^1^H NMR metabolomic profiles of plasma lipid molecules are correlated with mortality in PAD patients [25]. Specifically, alterations in lipoprotein and phospholipid structures were the major chemical signals that were distinct between PAD patients who died in the near-term versus those PAD patients who did not. The second study demonstrated that tyrosine and oxidized low-density lipoprotein (oxLDL) are associated with arterial stiffness in PAD patients [26]. These two seminal studies point to the potential of metabolomic profiling for providing significant insight into the pathophysiology of PAD. With this study, we aimed to expand the metabolomic mapping of PAD and to compare the circulating metabolites of patients with IC, patients with CLI, and non-PAD controls.

## 2. Materials and Methods

### 2.1. Study Approval and Subjects

Twenty-six non-PAD controls, 26 patients with IC, and 26 patients with CLI were recruited by vascular surgeons at the University of Nebraska Medical Center (UNMC, 00707), the Veterans Affairs Nebraska-Western Iowa Medical Center, and Baylor Scott and White Hospital (BSWI, 160390) under approved IRB protocols. IC or CLI diagnoses were made after examination of medical history, a physical examination, measurement of the ankle-brachial index (ABI), and computerized or standard arteriography. All non-PAD controls had normal blood flow to their legs and were undergoing operations for manifestations other than PAD (Table 1). These patients also had no history of PAD symptoms, normal lower limb pulses, normal ABIs at rest and after stress, were sex-matched, and all led sedentary lifestyles.

### 2.2. Sample Collection and Preparation

Blood samples were obtained in the morning after an overnight fast. In total, 30 mL of blood was obtained from each patient and was immediately centrifuged for 10 minutes, 2000 × *g* at 4 °C. Serum was aliquoted into separate polypropylene tubes and immediately stored at −80 °C. The Biocrates AbsoluteIDQ p400 HR kit (Biocrates Life Science AG, Innsbruck, Austria) was used to analyze 100 µL of serum from each patient. Compared to manual liquid chromatography-tandem mass spectrometry (LC-MS/MS) (Applied Biosystems/MDS Sciex., Foster City, CA, USA)) analysis and other technologies, the Biocrates kit allows for higher efficiency of separation, better limits of detection, decreased consumption of solvents, and absolute quantification of metabolites [27].

### 2.3. Targeted Identification and Quantification

The Biocrates AbsoluteIDQ p400 HR kit was used to measure more than 400 metabolites, including 21 amino acids, 21 biogenic amines, the sum of hexoses (as one metabolite, primarily glucose), 55 acylcarnitines, 18 diglycerides, 42 triglycerides, 24 lysophosphatidylcholines, 172 phosphatidylcholines, 31 sphingomyelins, 9 ceramides, and 14 cholesteryl esters. The complete list of metabolites assayed are provided in the Supplementary Material. Serum samples, blanks, calibration standards, and quality controls were prepared according to manual instructions. LC-MS/MS was used to analyze the amino acids and biogenic amines, and the remaining metabolites were analyzed by flow injection analysis (FIA) coupled with tandem mass spectrometry. All amino acids and biogenic amines were derivatized with phenylisothiocyanate. Metabolites were quantified using internal standards and multiple reactions monitoring (MRM). The samples were analyzed on a Thermo Scientific UltiMate 3000 Rapid Separation Quaternary HPLC System (Thermo Scientific, Madison, WI, USA), connected to a QExactive™ Focus Hybrid Quadrupole-Orbitrap™ Mass Spectrometer (Thermo Scientific, Waltham, MA, USA). The chromatographic column was obtained from Biocrates. Data first underwent a pre-processing step of peak integration to determine concentration based on calibration curves using Multiquant software (version 3.0, AB Sciex, Darmstadt, Germany). Following, data were uploaded into Biocrates MetIDQ software, and concentrations of FIA-monitored metabolites were calculated in MetIDQ. The experiment was also validated using the Biocrates software (version 5, MetIDQ, Biocrates, Innsbruck, Austria).

### 2.4. Phenylalanine/Tyrosine Ratio and Cholesteryl Esters CE (18:1)/CE (18:2)

We calculated the ratio of phenylalanine to tyrosine as a marker of inflammation [28] and the ratio of cholesteryl esters CE (18:1)/CE (18:2) as an indicator of the ratio of acyl-coenzyme A (CoA) cholesterol acyltransferase (ACAT), a pro-atherogenic enzyme, to serum lecithin cholesterol acyltransferase (LCAT), an anti-atherogenic enzyme that facilitates reverse cholesterol transport [29]. This relationship is based on the observation that CE (18:1) is the preferred fatty acid of ACAT, and CE (18:2) is preferred by LCAT. A higher (18:1) to (18:2) ratio is therefore believed to suggest higher ACAT activity [30,31].

### 2.5. Statistical Analyses

Baseline characteristics between non-PAD control, IC, and CLI subjects were compared using Chi-square and Fisher exact tests for categorical variables and analysis of variance (ANOVA) for continuous variables. One-way analysis of covariance (ANCOVA) was used for the rest of the analyses controlling for age, ABI, and diabetes mellitus. MetaboAnalyst 4.0 (www.metaboanalyst.ca) (version 4, McGill University, Montreal, QC, Canada) was used for statistical analysis of the metabolites’ data, processed with normalization, scaling, and filtering [32].

A one-way analysis of covariance was used to identify differences between non-PAD control, IC, and CLI groups for all the metabolites adjusting for any significant covariates, followed by *post-hoc* analyses with Bonferroni correction. Pearson correlations were calculated to evaluate associations between the ABI and the metabolites. Discriminant function models were developed to classify patients as non-PAD control, IC, or CLI. First, discriminant analysis assumptions were verified, and the multivariate data were standardized to remove units and place each variable on the same scale. A stepwise selection procedure was used to analyze variable contribution [33]. This method uses both forward selection and backward elimination procedures to determine the contribution of parameters to the discriminatory power of the model. Further, after derivation of a discriminant model, the model was used to classify new observations. A full cross-validation procedure was executed to evaluate the model performance. Cross-validation is a standard multivariate statistical method used on small data sets, validating the model by assessing stability and determining how well it will perform on other data sets [33]. During training, the cross-validation technique rotates the membership of the metabolite, verifying that the results are not dependent on calibration versus validation group membership, thus ensuring that the model is not overfitting the data. All analyses were performed using SAS statistical software (version 9.3, SAS Institute Inc., Cary, NC, USA).

## 3. Results

### 3.1. Patient Demographics 

The baseline demographic and clinical characteristics are presented in Table 1. As expected, IC and CLI patients had significantly lower ABI values than non-PAD control subjects (IC: 0.51 ± 0.18 vs. CLI: 0.18 ± 0.10 vs. non-PAD controls: 1.05 ± 0.05, *p* < 0.001). IC patients were younger than CLI patients (*p* = 0.044) and CLI patients had a higher ratio of diabetes mellitus (*p* = 0.005). No other differences were found among the different groups of subjects.

### 3.2. Amino Acids

Nineteen amino acids were measured for all subjects, and the phenylalanine/tyrosine ratio was also calculated. The amino acids arginine, glutamine, proline, tryptophan, and tyrosine were significantly lower in CLI patients when compared to both IC patients and non-PAD controls (Table 2). In addition, levels of histidine and ornithine were significantly lower in both CLI and IC patients compared to non-PAD controls. The phenylalanine/tyrosine ratio was significantly higher in both CLI and IC patients compared to non-PAD controls (Table 2). No other differences were observed.

Of the amino acids, ABI was significantly correlated with histidine (*r* = 0.463, *p* < 0.001), ornithine (*r* = 0.277, *p* = 0.017), tryptophan (*r* = 0.451, *p* < 0.001), and the phenylalanine/tyrosine ratio (*r* = −0.428, *p* < 0.001).

### 3.3. Acylcarnitines, Hexoses, and Biogenic Amines

Acycarnitine was significantly lower in CLI patients compared to IC patients and non-PAD controls. Acylcarnitne was also associated with the ABI (*r* = 0.378, *p* = 0.001). In contrast, hydroxypropionylcarnitine, propionylcarnitine, and tiglylcarnitine were significantly higher in both the IC and CLI patients compared to non-PAD controls (Table 3). Of the biogenic amines, only putrescine was significantly different, higher in CLI patients compared to both IC patients and non-PAD controls (Table 4). No other differences were observed.

### 3.4. Ceramides, Cholesteryl Esters, Sphingomyelins, Diglycerides, Triglycerides, and Phosphatidylcholines

Ceramides (Cer) (40:1), (41:1), and (42:1) were significantly lower in CLI patients compared to IC patients and non-PAD controls (Table 5). Cer (43:1) and (44:0) were significantly lower in both CLI and IC patients compared to non-PAD controls. Cholesteryl esters (CE) (16:0), (17:1), (18:2), (19:2), and (20:4) were significantly lower in CLI patients compared to IC patients and non-PAD controls (Table 6). Additionally, CE (17:0) was significantly lower in both CLI and IC patients compared to non-PAD controls, and the ratio of CE (18:1)/CE (18:2) was significantly higher in both CLI and IC patients compared to non-PAD controls. (Table 6). Finally, sphingomyelins, lysophosphatidylcholines, and phosphatidylcholines were all significantly lower in CLI patients compared to both IC patients and non-PAD controls (Table 6).

Of the ceramides, ABI was significantly associated with Cer (40:1) (*r* = 0.505, *p* < 0.001), Cer (41:1) (*r* = 0.541, *p* < 0.001), Cer (42:1) (*r* = 0.488, *p* < 0.001), and Cer (43:1) (*r* = 0.638, *p* < 0.001). Of the CEs, ABI was significantly associated with CE (16:0) (*r* = 0.374, *p* = 0.001), CE(17:0) (*r* = 0.482, *p* < 0.001), CE (17:1) (*r* = 0.446, *p* < 0.001), CE (18:2) (*r* = 0.447, *p* < 0.001), and CE (20:5) (*r* = 0.459, *p* < 0.001) (Table 7).

### 3.5. Discriminant Function Analysis: Non-PAD Control vs. PAD and IC vs. CLI

**Figure 1:**
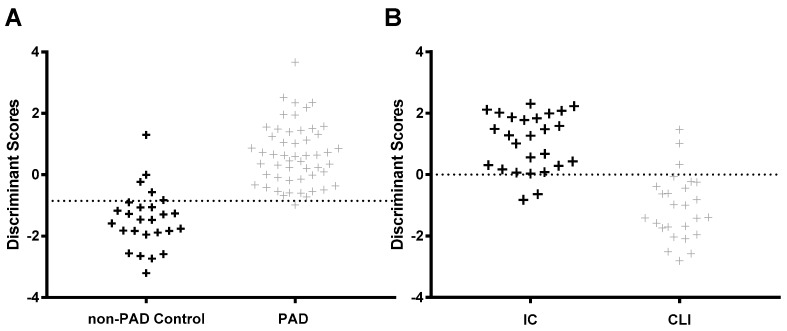
Discriminant function analysis model. Note: On the basis of metabolomic parameters, patient discriminant function scores can separate non-peripheral artery disease (PAD) control from PAD patients (**A**), and intermittent claudication (IC) patients from critical limb ischemia (CLI) patients (**B**).

The discriminant function analysis model was able to correctly classify the non-PAD control and PAD patients with a 93.6% accuracy. Using a cross-validation procedure to evaluate the discriminant model performance and stability also yielded an 87.2% accuracy in patient classification. Sensitivity and specificity are two basic quantities for measuring the accuracy of a diagnostic test. The sensitivity of a diagnostic test quantifies its ability to correctly identify non-PAD control subjects without the disease and is a measure of how well the test detects non-PAD control subjects. The sensitivity of the analysis was 73.1%. The specificity refers to the ability of a test to correctly identify subjects with PAD. The specificity of the analysis was 94.2%. Figure 1A represents the plot of the original discriminant function scores, and Equation (1) represents the unstandardized canonical discriminant function coefficients:

(1) $$\begin{array}{c} Di=-2.539-0.057\left(Carnitine\right)+243.9\left(Hydroxypropionylcarnitine\right)\\ \;-449.2\left(Propionylcarnitine\right)+1.36\left(\frac{Phenylalanine}{Tyrosine}\right)\\ \;+0.006\left(Alanine\right)-0.020\left(Glutamate\right). \end{array}$$

The discriminant function analysis model correctly classified the IC and CLI patients with a 90.4% accuracy. Using a cross-validation procedure to evaluate the discriminant model performance and stability also yielded an 84.6% accuracy in patient classification. For this model, the sensitivity (correctly identify IC patients) was 80.8%, and the specificity (correctly identify CLI patients) was 88.5%. Figure 1B represents the plot of the original discriminant function scores, and Equation (2) represents the unstandardized canonical discriminant function coefficients:

(2) $$\begin{array}{c} Di=-3.346+0.032\left(Histidine\right)+5.83\left(Butyrylcarnitine\right)+0.051\left(Tryptophan\right)\\ \;-0.865\left(Kynurenine\right). \end{array}$$

## 4. Discussion

We conducted a broad metabolomic profiling of small molecules and lipids and compared metabolites between patients with IC, patients with CLI, and non-PAD controls. To our knowledge, this is the first example of metabolomics applied to evaluate patients in the two symptomatic categories of PAD versus controls. Compared to non-PAD controls, we found significant changes in the circulating levels of multiple metabolites in patients with CLI and patients with IC (Table 8). Our discriminant function analysis was able to correctly classify subjects into symptomatic PAD (both IC and CLI) vs. non-PAD controls, as well as IC vs. CLI groups, with a 93.6% and 87.2% accuracy, respectively. We found that a series of metabolites or metabolite ratios, including histidine, ornithine, phenylalanine/tyrosine, hydroxypropionyl-carnitine, propenoylcarnitine, tiglylcarnitine, Cer (43:1), Cer (44:0), CE (17:0), and CE (18:1)/CE (18:2), are significantly different in symptomatic PAD patients compared to non-PAD controls (Table 8). Several of these metabolites were also significantly correlated with the ABI, a test which may indicate PAD severity. These variables may therefore prove useful as diagnostic markers to identify PAD patients among a large population [34]. The measurement of some or a combination of these metabolites, although not currently common in most clinical laboratories, could become a much-needed, standard test for the early detection of PAD and may be incorporated into routine care cardiovascular risk prediction [35]. Additionally, the levels of a number of metabolites or metabolite ratios, including arginine, glutamine, proline, tryptophan, tyrosine, acylcarnitine, putrescine, Cer (40:1), Cer (41:1), Cer (42:1), CE (16:0), CE (17:1), CE (18:2), CE (19:2), CE (20:4), sphingomyelins, phosphatidylcholines, and lysophosphatidylcholines, were significantly different in CLI patients compared to IC patients and may prove to be valuable biomarkers for indicating which patients with IC are at higher risk of progressing to CLI. The moderate correlations between several of the ceramides and the ABI further support this. Notably, the strongest association between any of the metabolites with the ABI was that for Cer (43:1) (*r* = 0.638). It is possible that these metabolites alone or in combination can be used to produce a high-risk profile for PAD patients, allowing a personalized approach (more aggressive management, earlier intervention, and more frequent follow up for higher risk patients and less aggressive for lower risk patients) to providing care for PAD patients. The utility of such a profile should be tested in the near future as it may be able to direct the general care of PAD patients.

**Figure 2:**
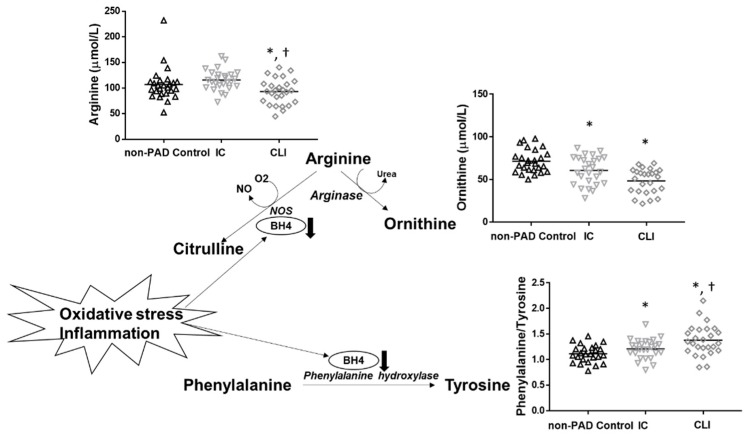
Potential mechanism (oxidative stress and inflammation) operating to produce decreased arginine and ornithine, increased phenylalanine to tyrosine ratio, and decreased nitric oxide bioavailability in PAD. Note: Tetrahydrobiopterin (BH4), an essential cofactor of nitric oxide synthase (NOS) and phenylalanine hydroxylase (PAH), is depleted by oxidative stress and inflammation. Therefore, reduced BH4 may explain the decreased turnover of phenylalanine to tyrosine observed in IC and CLI patients. Reduced BH4, as well as a lack of arginine availability, may play a role in impairing production of NO in PAD patients, leading to endothelial dysfunction. * denotes a significant difference from non-PAD controls and † denotes a significant difference from IC.

Several of the metabolite changes observed in this study in PAD patients are consistent with other reports from different pathologies. For example, serum levels of arginine were significantly reduced in CLI, which is consistent with several disorders linked to nitric oxide (NO) deficiency [36,37,38]. Likewise, ornithine, a byproduct of arginine, was reduced in both IC and CLI patients. Since NO is synthesized from *L*-arginine, lack of availability of this substrate is believed to be a factor that can lead to decreased plasma NO [36]. Impaired endothelial production of NO has been demonstrated in IC patients, and this may in fact be worse in patients with CLI [39]. Further, the ratio between serum levels of phenylalanine and tyrosine was elevated in PAD patients (both IC and CLI), and the phenylalanine/tyrosine ratio was inversely correlated with the ABI (*r* = −0.428). An elevated phenylalanine/tyrosine ratio has similarly been shown in different conditions associated with oxidative stress and inflammation, including acute brain ischemia [28]. This increased ratio may suggest diminished activity of the phenylalanine hydroxylase (PAH) enzyme by oxidation as well as tetrahydrobiopterin (BH4) deficiency, an essential cofactor of PAH [28]. Since BH4 is also a cofactor of nitric oxide synthase (NOS) and can be depleted by oxidative stress and inflammation, this ratio may also be indicative of NO dysregulation [39,40] (Figure 2**)**. Notably, oxidative stress and inflammation are hallmarks of PAD [41,42,43,44]. Several biomarkers of oxidative stress and inflammation, including malondyaldheide (MDA), 4-hydroxynonenale (4-HNE), isoprostanes, protein carbonyl groups, C-reactive protein (CRP), fibrinogen, tumor necrosis factor alpha (TNF-α), interferon-gamma (IFN-γ), monocyte chemoattractant protein-1 (MCP-1), and interleukin 6 (IL-6), have all been shown to be elevated in PAD patients in both circulation and skeletal muscle, and to increase with increasing disease stage [45].

PAD patients also demonstrated significantly reduced levels of histidine, an amino acid with antioxidant and anti-inflammatory properties [46]. In vitro, histidine has been shown to blunt pro-inflammatory cytokine expression, and histidine supplementation has been used to control inflammation in obese patients with metabolic syndrome [47]. Patients with different conditions of enhanced oxidative stress, such as chronic kidney disease and coronary heart disease, have also been shown to have reduced levels of histidine [46,48], suggesting that depletion of histidine may indicate elevated oxidative stress, a condition that is well described in patients with IC and CLI [41,49,50].

**Figure 3:**
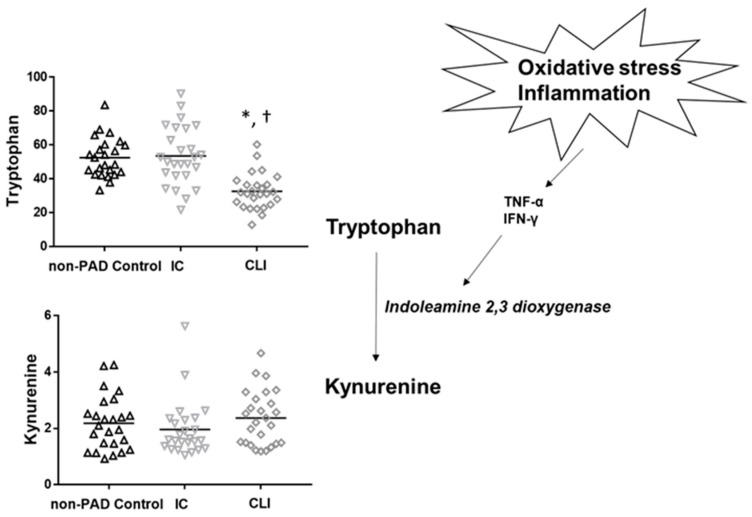
Potential mechanism (oxidative stress and inflammation) operating to produce decreased tryptophan levels in CLI. Note: Accelerated conversion of tryptophan to kynurenine is induced by inflammatory cytokines. Tryptophan is converted to kynurenine by the enzyme indoleamine 2,3 dioxygenase (IDO). IDO expression and activity are enhanced by tumor necrosis factor-alpha (TNF-α) and interferon-gamma (IFN-γ). Elevations in these cytokines may explain an increased conversion of tryptophan to kynurenine in CLI patients. * denotes a significant difference from non-PAD controls and † denotes a significant difference from IC.

CLI patients demonstrated further perturbations in amino acids not observed in IC patients that are also consistent with reports from other diseases and disorders. For example, reduced tryptophan levels have been associated with inflammation and immune activation, and have been shown to predict higher mortality in cardiovascular disease [51]. Specifically, reduced tryptophan may be due to accelerated conversion to kynurenine by indoleamine 2,3-dioxygenase (IDO1), which is activated by cytokines, such as tumor necrosis factor-alpha (TNF-α) and interferon gamma (IFN-γ) (Figure 3) [52]. Levels of kynurenine were also higher in CLI patients compared to both ICs and non-PAD controls, although differences were not significant statistically. In addition, glutamine levels were significantly lower in CLI patients. Reduced levels of glutamine are thought to be indicative of skeletal muscle catabolism [53]. Interestingly, CLI patients exhibit a severe myopathy that is characterized by myofiber degeneration, fibrosis, and muscle atrophy [49].

Several carnitine esters and members of the acyl carnitines, including hydroxypropionylcarnitine, propionylcarnitine, and tiglylcarnitine, were significantly elevated in both IC and CLI patients compared to non-PAD controls. During the metabolism of amino acids, carbohydrates, and fatty acids, these substrates are converted to acyl-CoA intermediates for oxidation in the Krebs cycle. Under functional metabolism, carnitine buffers acyl-CoA by forming acylcarnitines. However, during metabolic stress, acyl-CoA is incompletely oxidized and accumulates, and transfer of the acyl group to carnitine thus leads to accumulation of acylcarnitine. Therefore, accumulation of acylcarnitines can be an indication of dysfunctional metabolism [54]. Early studies in IC patients showed that short-chain acylcarnitines accumulate in plasma, which is inversely correlated with exercise performance [55]. In skeletal muscle tissue from patients with unilateral claudication, acylcarnitine accumulation was specific only to the affected limb, and interestingly, accumulation of acylcarnitine was shown to be a better indicator of exercise performance than even the ABI [56].

In patients with more severe PAD, however, a reduction in total and acylcarnitine content has been shown [57]. This is consistent with our study, in which CLI patients also demonstrated reduced total acylcarnitine levels, which has been thought to suggest dysfunctional fatty acid β-oxidation [58,59]. Additionally, in our study there was a weak positive association between the ABI and total acylcarnitine (*r* = 0.378). The rate-limiting step in the β-oxidation of long-chain fatty acids is the conjugation of carnitine to fatty acyl coenzyme A (coA) by the enzyme carnitine palmitoyltransferase (CPT1) [60]. Since acylcarnitine levels remain constant and only levels of free carnitine are affected by factors, such as age and sex, low acylcarnitine levels may suggest metabolic alterations due to decreased levels of total carnitine, reduced CPT1 activity, or decreased availability of acyl-coA [59]. Consistent with a potentially dysfunctional fatty acid β-oxidation, muscle tissue from CLI patients demonstrates reduced expression of oxidative phosphorylation proteins, as well as lower mitochondrial respiratory capacity [15]. However, other carnitine esters, including hydroxypropionylcarnitine, propionylcarnitine, and tigylcarnitine, were significantly elevated in both IC and CLI patients compared to non-PAD controls. Therefore, the role of carnitines in PAD warrants further exploration.

Sphingolipids are major components of cellular membranes, critical for the fluidity and architecture of the membrane. Sphingomyelins are a type of sphingolipid usually consisting of phosphocholine and ceramide. The metabolites of sphingolipids, such as ceramides, are important for regulating cell proliferation and survival, as well as the inflammatory responses [61]. In this study, levels of certain ceramides were markedly reduced in both IC and CLI patients. Further, CLI patients demonstrated reduced levels of sphingomyelins. Similar findings were reported for patients with sickle-cell disease, which is associated with a progressive vasculopathy, vascular occlusion, and endothelial dysfunction, all of which are pathophysiological aspects of PAD as well [39,62]. In contrast, however, other studies have shown that sphingomyelin and ceramides are independent risk factors for coronary heart disease and that higher levels are associated with atherosclerosis and the development of metabolic disease [63,64,65,66]. Future research is needed to clarify the alternations in sphingolipid metabolism in CLI patients. Phosphatidylcholine levels were lower in CLI patients, which is consistent with a study in patients with atherosclerosis, where reduced phosphatidylcholine levels were correlated with increased arterial stiffness, increased resting heart rate, and/or worsened endothelial function [67]. Interestingly, the hydrolysis of phosphatidylcholine to phosphatidic acid and choline is catalyzed by phospholipase D (PLD), and high PLD activity is associated with oxidative stress, inflammation, hypoxia, and atherosclerosis [68]. Finally, several cholesteryl ester species were lower in CLI patients as well. Of note, the ratio of cholesteryl ester CE (18:1)/CE (18:2) was significantly increased in both IC and CLI patients, which is consistent with findings in high fat diet-induced obese mice [29]. Since CE (18:1) is considered the preferred fatty acid of ACAT, this suggests higher ACAT activity, which is consistent with obesity and hypercholesterolemia [30].

Currently available options (risk factor management, medications, exercise therapy, and revascularization operations) for the management of PAD are limited. There are two medications, with only modest efficacy, approved for claudication, and operations are associated with considerable morbidity and poor durability [69,70]. PAD patients suffer from high rates of cardiovascular events, including stroke and myocardial infarction [71], and PAD also significantly impairs quality of life and leads to functional impairment and decline [72]. This highlights the importance of identifying novel targets for intervention for this population. In this study, we identified several metabolites that are altered in symptomatic PAD patients compared to non-PAD controls while also identifying a distinct metabolomic signature associated with only CLI [15].

One important limitation of this study is the sample size was relatively small. Thus, external validation from a larger sample could help add to the translational impact of the study results. Furthermore, while there is an emerging use of metabolomics in clinical settings, complexities and challenges, for example, related to testing strategies and quality control, limit its immediate clinical impact. Thus far, the field of metabolomics has primarily been limited to biomedical research and biomarker discovery; however, greater considerations must be taken into account for use as a clinical test. Therefore, this may also affect the translational impact of this study.

Another important point to note is the use of only serum may limit the generalizability of these findings to other fluids and tissues. Specifically, during centrifugation to separate serum from coagulated blood, platelets release proteins that include cytokines and metabolites into the serum [73]. Since anticoagulants are added before the removal of blood cells to obtain plasma, there may be differences between human plasma and serum metabolites. However, in a large study that compared metabolite concentrations between plasma and serum, although there were differences in the exact concentrations between blood matrices, the changes between groups were proportional and the correlation was high between plasma and serum [73]. This study also concluded that reproducibility was high in both plasma and serum and that either will lead to similar results in clinical studies (as long as the same matrix is used throughout), with serum potentially providing greater sensitivity in biomarker studies, thus supporting our use of serum in this study [73].

## 5. Conclusions

In conclusion, we identified a number of metabolites that are altered in PAD. To our knowledge, this is the first time that a complete metabolomic profiling comparing patients with different severities of PAD and non-PAD controls is presented. These data provide unique metabolomic fingerprints that may be helpful in screening for the presence of PAD, and may also be useful in risk-stratifying PAD patients and predicting their clinical outcomes. Further, these alterations provide insight into the disrupted pathways that underlie the pathophysiology of PAD and may contribute to a better understanding of the disease and to the development of novel therapeutic interventions for PAD patients.

## Figures and Tables

**Table 1:** Patient demographics at enrollment. Data are shown as mean ± standard deviation.

	Non-PAD Control (*n* = 26)	IC (*n* = 26)	CLI (*n* = 26)	*p*
Age (years)	63.2 ± 7.4	62.0 ± 7.3	67.6 ± 9.9 ^†^	**0.044**
Male sex (%)	23 (88.50)	24 (92.3)	26 (100)	0.224
Body mass index	29.6 ± 6.5	27.1 ± 9.9	27.8 ± 5.6	0.476
ABI	1.05 ± 0.05	0.51 ± 0.18 *	0.18 ± 0.10 *^,†^	**<0.001**
**Risk factors (%)**				
Tobacco use				0.073
Current	10 (38.5)	14 (53.8)	6 (23.1)	
Never	9 (34.6)	3 (11.5)	6 (23.1)	
Former	7 (26.9)	9 (34.6)	14 (53.8)	
Hypertension	17 (65.4)	22 (84.6)	23 (88.5)	0.087
Diabetes mellitus	4 (15.4)	8 (30.8)	15 (57.7) *^,†^	**0.005**
Coronary Artery Disease	9 (34.6)	13 (50.0)	16 (61.5)	0.150
Obesity	9 (34.6)	7 (26.9)	6 (23.1)	0.642
Dyslipidemia	21 (80.8)	19 (73.1)	16 (61.5)	0.300

Note: The values presented in the column “*p*-value” represent the overall difference between the three groups; bold font indicates a significant difference between groups (*p* < 0.05); post-hoc differences in comparisons between individual groups are denoted below as: * = significant difference from non-PAD control, *p* < 0.05; † = significant difference from IC, *p* < 0.05.

**Table 2:** Serum amino acids concentrations of the study subjects. Data are shown as mean ± standard error (μmol/L).

	Non-PAD Control (*n* = 26)	IC (*n* = 26)	CLI (*n* = 26)	*p*
Alanine	291.3 ± 23.1	356.1 ± 22.6 *	258.6 ± 24.0 †	**0.014**
Arginine	109.1 ± 5.3	117.4 ± 5.2	89.1 ± 5.5 *^,†^	**0.002**
Asparagine	31.7 ± 2.1	34.1 ± 2.0	30.5 ± 2.2	0.482
Citrulline	32.7 ± 3.6	37.5 ± 3.6	34.8 ± 3.8	0.626
Glutamine	580.5 ± 20.2	583.8 ± 19.8	495.3 ± 20.9 *^,†^	**0.007**
Glutamate	88.0 ± 5.9	65.7 ± 5.8 *	72.7 ± 6.2	***0.029***
Glycine	271.4 ± 20.1	298.6 ± 19.7	275.1 ± 20.9	0.575
Histidine	80.4 ± 2.7	73.0 ± 2.6 *	54.6 ± 2.8 *^,†^	***<*0.001**
Leucine	199.5 ± 12.6	203.5 ± 12.4	177.6 ± 13.1	0.352
Lysine	146.2 ± 9.2	143.3 ± 8.9	121.9 ± 9.1	0.165
Methionine	30.8 ± 7.3	50.8 ± 7.2	30.4 ± 7.6	0.083
Ornithine	70.1 ± 3.9	60.7 ± 3.9 *	53.3 ± 4.2 *	**0.016**
Phenylalanine	70.1 ± 3.7	69.7 ± 3.6	64.6 ± 3.9	0.567
Proline	207.2 ± 11.3	224.4 ± 11.9	158.9 ± 11.7 *^,†^	**0.001**
Serine	120.4 ± 4.9	117.7 ± 4.8	105.5 ± 5.1	0.116
Threonine	122.9 ± 8.3	121.6 ± 8.2	108.8 ± 8.6	0.473
Tryptophan	48.9 ± 3.1	53.6 ± 3.1	32.5 ± 3.2 *^,†^	**<0.001**
Tyrosine	62.7 ± 3.2	58.1 ± 3.2	46.6 ± 3.4 *^,†^	**0.006**
Valine	72.9 ± 5.1	80.6 ± 5.5	71.1 ± 5.3	0.376
Phenylalanine/Tyrosine	1.05 ± 0.06	1.25 ± 0.06 *	1.45 ± 0.07 *^,†^	**0.001**

Note: The values presented in the column “*p*-value” represent the overall difference between the three groups; bold font indicates a significant difference between groups (*p* < 0.05); post-hoc differences in comparisons between individual groups are denoted below as: * = significant difference from non-PAD control, *p* < 0.05; ^†^ = significant difference from IC, *p* < 0.05.

**Table 3:** Concentrations of serum acylcarnitines and hexoses of the study subjects. Data are shown as mean ± standard error (μmol/L).

	Non-PAD Control (*n* = 26)	IC (*n* = 26)	CLI (*n* = 26)	*p*
Acylcarnitine	43.5 ± 2.1	40.6 ± 2.1	31.1 ± 2.2 *^,†^	**0.001**
Acetyl-L-carnitine	8.7 ± 1.0	9.5 ± 1.1	9.6 ± 1.1	0.816
Propionylcarnitine	0.351 ± 0.038	0.391 ± 0.037	0.294 ± 0.039	0.221
Malonylcarnitine	0.006 ± 0.001	0.006 ± 0.001	0.007 ± 0.001	0.452
Hydroxypropionylcarnitine	0.040 ± 0.002	0.046 ± 0.002 *	0.047 ± 0.002 *	**0.003**
Propenoylcarnitine	0.017 ± 0.001	0.019 ± 0.001 *	0.019 ± 0.001 *	**0.050**
Butyrylcarnitine	0.169 ± 0.023	0.168 ± 0.023	0.153 ± 0.024	0.869
Hydroxybutyrylcarnitine	0.055 ± 0.015	0.093 ± 0.015	0.084 ± 0.016	0.189
Butenylcarnitine	0.030 ± 0.001	0.030 ± 0.001	0.029 ± 0.001	0.879
Isovalerylcarnitine	0.086 ± 0.01	0.092 ± 0.01	0.066 ± 0.01	0.189
Tiglylcarnitine	0.023 ± 0.01	0.025 ± 0.01 *	0.026 ± 0.01 *	**0.038**
Hexoses	5266 ± 489	6034 ± 479	5967 ± 508	0.476

Note: The values presented in the column “*p*-value” represent the overall difference between the three groups; bold font indicates a significant difference between groups (*p* < 0.05); post-hoc differences in comparisons between individual groups are denoted below as * = significant difference from non-PAD control, *p* < 0.05; † = significant difference from IC, *p* < 0.05.

**Table 4:** Concentrations of serum biogenic amines of the study subjects. Data are shown as mean ± standard error (μmol/L). ADMA: Asymmetric dimethyl arginine. SDMA: Symmetric dimethyl arginine. Met-SO: Methionine sulfoxide. t4-OH-Pro: Trans-4-hydroxyproline.

	Control (*n* = 26)	IC (*n* = 24)	CLI (*n* = 24)	*p*
ADMA	0.549 ± 0.03	0.543 ± 0.03	0.591 ± 0.03	0.544
SDMA	0.491 ± 0.05	0.530 ± 0.04	0.614 ± 0.05	0.249
Creatinine	92.8 ± 19.6	103.1 ± 19.5	139.1 ± 19.5	0.260
Kynurenine	2.03 ± 0.203	1.90 ± 0.199	2.42 ± 0.211	0.211
Met-SO	1.35 ± 0.847	3.47 ± 0.830	2.35 ± 0.880	0.198
Putrescine	0.011 ± 0.063	0.059 ± 0.062	0.328 ± 0.066 *^,†^	**0.003**
Serotonin	0.503 ± 0.107	0.793 ± 0.089	0.769 ± 0.114	0.096
Spermide	0.453 ± 0.137	0.312 ± 0.134	0.440 ± 0.142	0.716
t4-OH-Pro	11.0 ± 1.68	10.8 ± 1.65	10.4 ± 1.64	0.938
Taurine	86.7 ± 10.1	88.3 ± 12.3	65.6 ± 12.2	0.370

Note: The values presented in the column “*p*-value” represent the overall difference between the three groups; bold font indicates a significant difference between groups (*p* < 0.05); post-hoc differences in comparisons between individual groups are denoted below as: * = significant difference from non-PAD control, *p* < 0.05; † = significant difference from IC, *p* < 0.05.

**Table 5:** Concentrations of serum ceramides of the study subjects. Data are shown as mean ± standard error (μmol/L).

	Control (*n* = 26)	IC (*n* = 24)	CLI (*n* = 24)	*p*
Cer (34:0)	0.054 ± 0.002	0.052 ± 0.002	0.045 ± 0.02*	**0.038**
Cer (34:1)	0.174 ± 0.011	0.182 ± 0.011	0.168 ± 0.012	0.664
Cer (38:1)	0.136 ± 0.01	0.129 ± 0.01	0.114± 0.01	0.356
Cer (40:1)	0.702 ± 0.05	0.688 ± 0.04	0.508 ± 0.05 *^,†^	**0.014**
Cer (41:1)	0.550 ± 0.04	0.530 ± 0.04	0.312 ± 0.04 *^,†^	**0.001**
Cer (42:1)	2.16 ± 0.151	2.31 ± 0.148	1.35 ± 0.156 *^,†^	**<0.001**
Cer (42:2)	1.39 ± 0.083	1.46 ± 0.081	1.19 ± 0.086	0.094
Cer (43:1)	0.555 ± 0.030	0.466 ± 0.030 *	0.300 ± 0.032 *^,†^	**<0.001**
Cer (44:0)	0.286 ± 0.026	0.207 ± 0.025 *	0.202 ± 0.027 *	**0.048**

Note: The values presented in the column “*p*-value” represent the overall difference between the three groups; bold font indicates a significant difference between groups (*p* < 0.05); post-hoc differences in comparisons between individual groups are denoted below as: * = significant difference from non-PAD control, *p* < 0.05; † = significant difference from IC, *p* < 0.05.

**Table 6:** Concentrations of serum cholesteryl esters (CE), sphingomyelins, diglycerides, triglycerides, and phosphatidylcholines of the study subjects. Data are shown as mean ± standard error (μmol/L).

	Control (*n* = 26)	IC (*n* = 24)	CLI (*n* = 24)	*p*
CE (16:0)	168.9 ± 10.3	160.2 ± 10.1	106.8 ± 10.7 *^,†^	**0.001**
CE (16:1)	96.6 ± 14.9	103.6 ± 14.6	69.4 ± 15.5	0.281
CE (17:0)	10.9 ± 0.56	9.4 ± 0.55 *	7.2 ± 0.58 *^,†^	**0.001**
CE (17:1)	8.82 ± 0.76	8.45 ± 0.75	5.37 ± 0.79 *^,†^	**0.007**
CE (17:2)	0.94 ± 0.16	0.85 ± 0.15	0.49 ± 0.16	0.152
CE (18:1)	244 ± 55	422 ± 54	302 ± 58	0.067
CE (18:2)	3,015± 176	2,791 ± 172	1,870 ± 183 *^,†^	**<0.001**
CE (18:3)	119 ± 12.3	123.2 ± 12.0	83.8 ± 12.7	0.069
CE (19:2)	5.58 ± 0.64	5.82 ± 0.63	2.69 ± 0.66 *^,†^	**0.001**
CE (19:3)	0.92 ± 0.266	1.86 ± 0.26 *	0.49 ± 0.277 ^†^	**0.002**
CE (20:4)	911 ± 70	892 ± 69	662 ± 73 *^,†^	**0.041**
CE (20:5)	91.9 ± 7.5	78.5 ± 7.4	59.7 ± 7.8 *	**0.022**
CE (22:5)	43.3 ± 2.5	46.2 ± 2.4	37.6 ± 2.6	0.069
CE (22:6)	91.3 ± 5.9	87.0 ± 5.8	71.4 ± 6.2	0.078
CE (18:1)/CE (18:2)	0.084 ± 0.02	0.169 ± 0.02 *	0.186 ± 0.02 *	**0.008**
Sphingomyelins	11.1 ± 0.49	10.8 ± 0.48	8.80 ± 0.51 *^,†^	**0.007**
Diglycerides	3.48 ± 0.34	4.47 ± 0.33 *	3.07 ± 0.348 ^†^	**0.014**
Triglycerides	29.1 ± 3.91	40.1 ± 3.83	27.8 ± 4.06	0.055
Lysophosphatidylcholines	4.13 ± 0.24	4.44 ± 0.24	2.66 ± 0.25 *^,†^	**0.001**
Phosphatidylcholines	10.2 ± 0.65	12.2 ± 0.63 *	8.38 ± 0.67 *^,†^	**0.001**

Note: The values presented in the column “*p*-value” represent the overall difference between the three groups; bold font indicates a significant difference between groups (*p* < 0.05); post-hoc differences in comparisons between individual groups are denoted below as: * = significant difference from non-PAD control, *p* < 0.05; † = significant difference from IC, *p* < 0.05.

**Table 7:** Pearson correlation between ankle brachial index (ABI) and metabolites.

Metabolite/Metabolite Ratio	Pearson Correlation Coefficient (*r*)	Significance (*p*)
Acylcarnitine	0.378	0.001
Histidine	0.463	<0.001
Ornithine	0.277	0.017
Trytophan	0.451	<0.001
Phenylalanine/Tyrosine	−0.428	<0.001
Cer (40:1)	0.505	<0.001
Cer (41:1)	0.541	<0.001
Cer (42:1)	0.488	<0.001
Cer (43:1)	0.638	<0.001
CE (16:0)	0.374	0.001
CE (17:0)	0.482	<0.001
CE (17:1)	0.446	<0.001
CE (18:2)	0.447	<0.001
CE (20:5)	0.459	<0.001

**Table 8:** List of altered metabolites in PAD.

Metabolite Class	Different in CLI	Higher/Lower	Different in PAD	Higher/Lower
**Amino Acids**	Arginine	↓	Histidine	↓
	Glutamine	↓	Ornithine	↓
	Proline	↓	Phenylalanine/tyrosine	↑
	Tryptophan	↓		
	Tyrosine	↓		
**Acylcarnitines**	Acylcarnitine	↓	Hydroxypropionyl-carnitine	↑
			Propenoylcarnitine	↑
			Tigylcarnitine	↑
**Biogenic amines**	Putrescine	↑		
**Ceramides**	Cer (40:1)	↓	Cer (43:1)	↓
	Cer (41:1)	↓	Cer (44:0)	↓
	Cer (42:1)	↓		
**Cholesteryl esters, Sphingomyelin phosphatidyl-cholines**	CE (16:0)	↓	CE (17:0)	↓
	CE (17:1)	↓	CE (18:1)/CE (18:2)	↑
	CE (18:2)	↓		
	CE (19:2)	↓		
	CE (20:4)	↓		
	Sphingomyelins	↓		
	Phosphatidyl-cholines	↓		
	Lysophosphatidyl-cholines	↓		

Note: “Different in CLI” are the metabolites or metabolite ratios that were significantly different between CLI patients and both IC patients and non-PAD controls. “Different in PAD” are the metabolites or metabolite ratios that were significantly different between both CLI and IC patients and non-PAD controls. ↑ represents higher and ↓ represents lower.

## Supplementary Materials

The following are available online at https://www.mdpi.com/2077-0383/8/9/1463/s1.
