# Supplementary material for: Altered Metabolomic Profile in Patients with Peripheral Artery Disease
Source: J Clin Med. 2019 Sep 14;8(9):1463. doi: 10.3390/jcm8091463 (PMC6780416; doi:10.3390/jcm8091463)
Supplement: Supplementary file 1 [file jcm-08-01463-s001.pdf]

Supplement 1. List of Metabolites Analyzed

Amino Acids

Alanine, Lysine, Arginine, Methionine, Asparagine, Ornithine, Aspartate, Phenylalanine, Citrulline, Proline, Glutamate, Serine, Glutamine, Threonine, Glycine, Tryptophan, Histidine, Tyrosine, Isoleucine, Valine

Biogenic Amines

Acetylornithine, Nitrotyrosine, Asymmetric dimethylarginine, Phenylethylamine, alpha-Aminoadipic acid, Putrescine, Carnosine, Sarcosine, cis-4-Hydroxyproline, Symmetric dimethylarginine, Creatinine, Serotonin, Dihydroxyphenylalanine, Spermidine, Dopamine, Spermine, Histamine, Taurine, Kynurenine, trans-4-Hydroxyproline, Methionine sulfoxide

Monosaccharides

Hexoses (including glucose)

Acylcarnitines

Carnitine, Fumarylcarnitine, Acetylcarnitine, Valerylcarnitine, Propionylcarnitine, Glutarylcarnitine, Malonylcarnitine, Hydroxyvalerylcarnitine, Hydroxypropionylcarnitine, Tiglylcarnitine, Propenoylcarnitine, Glutaconylcarnitine, Butyrylcarnitine, Hexanoylcarnitine, Methylmalonylcarnitine, Adipoylcarnitine, Hydroxybutyrylcarnitine, Hydroxyhexanoylcarnitine, Butenylcarnitine, Hexenoylcarnitine, Heptanoylcarnitine, Carboxytridecenoylcarnitine, Pimeloylcarnitine, Hydroxytetradecenoylcarnitine, Octanoylcarnitine, Tetradecadienoylcarnitine, Octenoylcarnitine, Hydroxytetradecadienoylcarnitine, Hydroxyoctenoylcarnitine, Pentadecanoylcarnitine, Nonaylcarnitine, Hexadecanoylcarnitine, Decanoylcarnitine, Hydroxyhexadecanoylcarnitine, Decenoylcarnitine, Hexadecenoylcarnitine, Decadienoylcarnitine, Hydroxyhexadecenoylcarnitine, Decatrienoylcarnitine, Hexadecadienoylcarnitine, Dimethylnonanoylcarnitine, Hydroxyhexadecadienoylcarnitine, Dodecanoylcarnitine, Heptadecanoylcarnitine, Dodecanedioylcarnitine, Octadecanoylcarnitine, Dodecenoylcarnitine, Octadecenoylcarnitine, Tridecanoylcarnitine, Hydroxyoctadecenoylcarnitine, Tetradecanoylcarnitine, Octadecadienylcarnitine, Hydroxymyristoylcarnitine, Nonadecanoylcarnitine, Tetradecenoylcarnitine

Diglycerides

DG(32:1), DG(36:3), DG(41:1), DG-O(32:2), DG(32:2), DG(36:4), DG(42:0), DG-O(34:1), DG(34:1), DG(38:0), DG(42:1), DG-O(36:4), DG(34:3), DG(38:5), DG(42:2), DG(36:2), DG(39:0), DG(44:3)

Triglycerides

TG(44:1), TG(50:3), TG(52:6), TG(54:7), TG(44:2), TG(50:4), TG(52:7), TG(55:6), TG(44:4), TG(51:1), TG(53:3), TG(55:7), TG(46:2), TG(51:2), TG(53:4), TG(55:8), TG(48:1), TG(51:3), TG(53:5), TG(55:9), TG(48:2), TG(51:4), TG(53:6), TG(56:6), TG(48:3), TG(51:5), TG(54:2), TG(56:7), TG(49:1), TG(52:2), TG(54:3), TG(56:8), TG(49:2), TG(52:3), TG(54:4), TG(56:9), TG(50:1), TG(52:4), TG(54:5), TG(50:2), TG(52:5), TG(54:6)

Lysophosphatidylcholines

LPC(12:0), LPC(17:1), LPC(20:2), LPC(24:1), LPC(14:0), LPC(18:0), LPC(20:3), LPC-O(16:1), LPC(15:0),

LPC(18:1), LPC(20:4), LPC-O(17:1), LPC(16:0), LPC(18:2), LPC(22:5), LPC-O(18:0), LPC(16:1), LPC(20:0), LPC(22:6), LPC-O(18:1), LPC(17:0), LPC(20:1), LPC(24:0), LPC-O(18:2)

Phosphatidylcholines

PC(24:0), PC(36:1), PC(41:5), PC-O(34:0), PC(25:0), PC(36:2), PC(41:8), PC-O(34:1), PC(26:0), PC(36:3), PC(42:0), PC-O(34:2), PC(27:0), PC(36:4), PC(42:1), PC-O(34:3), PC(27:1), PC(36:5), PC(42:2), PC-O(34:4), PC(28:1), PC(36:6), PC(42:3), PC-O(35:3), PC(29:0), PC(37:0), PC(42:4), PC-O(35:4), PC(29:1), PC(37:1), PC(42:5), PC-O(36:0), PC(29:2), PC(37:2), PC(42:6), PC-O(36:1), PC(30:0), PC(37:3), PC(42:7), PC-O(36:2), PC(30:1), PC(37:4), PC(42:10), PC-O(36:3), PC(30:2), PC(37:5), PC(43:2), PC-O(36:4), PC(30:3), PC(37:6), PC(43:6), PC-O(36:5), PC(31:0), PC(37:7), PC(44:1), PC-O(36:6), PC(31:1), PC(38:0), PC(44:3), PC-O(37:6), PC(31:2), PC(38:1), PC(44:5), PC-O(37:7), PC(31:3), PC(38:2), PC(44:6), PC-O(38:0), PC(32:0), PC(38:3), PC(44:7), PC-O(38:1), PC(32:1), PC(38:4), PC(44:10), PC-O(38:2), PC(32:2), PC(38:5), PC(44:12), PC-O(38:3), PC(32:3), PC(38:6), PC(46:1), PC-O(38:4), PC(32:4), PC(38:7), PC(46:2), PC-O(38:5), PC(32:5), PC(39:0), PC-O(26:0), PC-O(38:6), PC(32:6), PC(39:1), PC-O(26:1), PC-O(40:0), PC(33:0), PC(39:2), PC-O(28:0), PC-O(40:1), PC(33:1), PC(39:3), PC-O(28:1), PC-O(40:2), PC(33:2), PC(39:4), PC-O(29:0), PC-O(40:3), PC(33:3), PC(39:5), PC-O(30:0), PC-O(40:4), PC(33:4), PC(39:6), PC-O(30:1), PC-O(40:5), PC(33:5), PC(39:7), PC-O(30:2), PC-O(40:6), PC(34:0), PC(40:1), PC-O(31:0), PC-O(40:7), PC(34:1), PC(40:2), PC-O(31:1), PC-O(40:8), PC(34:2), PC(40:3), PC-O(31:3), PC-O(42:0), PC(34:3), PC(40:4), PC-O(32:0), PC-O(42:1), PC(34:4), PC(40:5), PC-O(32:1), PC-O(42:2), PC(34:5), PC(40:6), PC-O(32:2), PC-O(42:3), PC(35:0), PC(40:7), PC-O(32:3), PC-O(42:4), PC(35:1), PC(40:8), PC-O(33:0), PC-O(42:5), PC(35:2), PC(40:9), PC-O(33:1), PC-O(42:6), PC(35:3), PC(41:1), PC-O(33:2), PC-O(44:3), PC(35:4), PC(41:2), PC-O(33:3), PC-O(44:4), PC(35:5), PC(41:3), PC-O(33:4), PC-O(44:5), PC(36:0), PC(41:4), PC-O(33:6), PC-O(44:6)

Sphingomyelins

SM(30:1), SM(34:2), SM(38:3), SM(42:1), SM(31:0), SM(35:1), SM(39:1), SM(42:2), SM(31:1), SM(36:0), SM(39:2), SM(42:3), SM(32:1), SM(36:1), SM(40:1), SM(43:1), SM(32:2), SM(36:2), SM(40:2), SM(43:2), SM(33:1), SM(37:1), SM(40:4), SM(44:1), SM(33:2), SM(38:1), SM(41:1), SM(44:2), SM(34:1), SM(38:2), SM(41:2)

Ceramides

Cer(34:0), Cer(40:1), Cer(42:2), Cer(34:1), Cer(41:1), Cer(43:1), Cer(38:1), Cer(42:1), Cer(44:0)

Cholesteryl Esters

CE(16:0), CE(17:2), CE(19:2), CE(22:5), CE(16:1), CE(18:1), CE(19:3), CE(22:6), CE(17:0), CE(18:2), CE(20:4), CE(17:1), CE(18:3), CE(20:5)
